# Violence Exposure Is Associated With Atypical Appraisal of Threat Among Women: An EEG Study

**DOI:** 10.3389/fpsyg.2020.576852

**Published:** 2021-01-12

**Authors:** Virginie Chloé Perizzolo Pointet, Dominik Andrea Moser, Marylène Vital, Sandra Rusconi Serpa, Alexander Todorov, Daniel Scott Schechter

**Affiliations:** ^1^Department of Psychiatry, Faculty of Medicine, University of Geneva, Geneva, Switzerland; ^2^University Service of Child and Adolescent Psychiatry, Lausanne University Medical Center, Lausanne, Switzerland; ^3^Institute of Psychology, University of Bern, Bern, Switzerland; ^4^Department of Child and Adolescent Psychiatry, University of Geneva Hospitals, Geneva, Switzerland; ^5^Department of Psychology, Faculty of Psychology and Educational Science, University of Geneva, Geneva, Switzerland; ^6^Department of Psychology, Princeton University, Princeton, NJ, United States; ^7^Department of Child and Adolescent Psychiatry, New York University Langone School of Medicine, New York, NY, United States

**Keywords:** microstates, source localization, IPV-PTSD, face evaluation, EEG neuroimaging

## Abstract

**Introduction:**

The present study investigates the association of lifetime interpersonal violence (IPV) exposure, related posttraumatic stress disorder (IPV-PTSD), and appraisal of the degree of threat posed by facial avatars.

**Methods:**

We recorded self-rated responses and high-density electroencephalography (HD-EEG) among women, 16 of whom with lifetime IPV-PTSD and 14 with no PTSD, during a face-evaluation task that displayed male face avatars varying in their degree of threat as rated along dimensions of dominance and trustworthiness.

**Results:**

The study found a significant association between lifetime IPV exposure, under-estimation of dominance, and over-estimation of trustworthiness. Characterization of EEG microstates supported that lifetime IPV-PTSD modulates emotional appraisal, specifically in encoding and decoding processing associated with N170 and LPP evoked potentials. EEG source localization demonstrated an overactivation of the limbic system, in particular the parahippocampal gyrus, in response to non-threatening avatars. Additionally, dysfunctional involvement of attention-related processing anterior prefrontal cortex (aPFC) was found in response to relatively trustworthy avatars in IPV-PTSD individuals compared with non-PTSD controls.

**Discussion:**

This study showed that IPV exposure and related PTSD modulate individuals’ evaluation of facial characteristics suggesting threat. Atypical processing of these avatar characteristics was marked by group differences in brain regions linked to facial processing, emotion regulation, and memory.

## Introduction

It is an essential social skill to be able to evaluate the human face in order to assess one’s level of safety in interacting with others. This requires the appraisal of an individual’s character traits, emotional states, and possible intentions, which in turn helps the observer to form a social judgment as to whether to engage in, refuse, and/or flee from further interaction. These types of social judgments can be formed within the very first milliseconds of facial evaluation and as early in human development as the first years of life (Chu et al., 2016). Some theorists have argued that social judgment or “appraisal” of a facial stimulus may be foremost a cognitive vs affective or physiologic (i.e., gut reaction) process, whereby the individual possibly for defensive or other reasons does not link associated emotional or physiological response to that of her cognitive judgment (Arnold and Gasson, 1954; Schachter and Singer, 1962). This latter notion is consistent with the Cannon–Bard theory of emotion (Cannon, 1927), whereby cognitive, affective, and physiological processes may be independent from one another and only more or less associated through brain circuitry involving the thalamus (Lang, 1994) when making a snap-judgment.

In evolutionary terms, such snap-judgments likely perform a key role in the detection of threat that can be necessary for survival, even if they are subject to error (Todorov et al., 2015). According to the literature, evaluating threat involves appraisal from a linear combination of perception along two basic dimensions: (1) “valence/trustworthiness” and (2) “power/dominance” (Oosterhof and Todorov, 2008; Todorov et al., 2008). The trustworthiness dimension involves sensitivity to features that would lead the observer to approach vs avoid a given individual. The dominance dimension involves sensitivity to features that would lead the observer to estimate whether a given individual is of relatively greater or lesser physical strength and potentially aggressive toward a subordinate. Dominance and trustworthiness appraisals when examining another person’s face can lead to inferences about that person’s intentions (i.e., harmful vs well-intended) as well as his or her ability to act on them. In the absence of clear emotional cues (i.e., emotionally neutral faces or other information), individuals infer others’ intentions based on assumptions about trustworthiness and dominance that are discerned from the evaluation of others’ faces (Montepare and Dobish, 2003; Todorov and Duchaine, 2008; Said et al., 2009; Zebrowitz et al., 2010). Threatening faces are perceived as both more dominant and less trustworthy. The degree of threat perceived thus corresponds empirically to the rating of a series of faces that vary in terms of the prominence of their characteristics of dominance and trustworthiness (Todorov et al., 2008).

Inferences, such as threat evaluation, could be positively and/or negatively affected by one’s own history of stressful interpersonal experiences and adverse life events, such as childhood maltreatment (Gibb et al., 2009; Nazarov et al., 2014; Neukel et al., 2019). Exposure to a hostile caregiving environment can lead to changes in emotional information processing and thus impact an individual’s perception of another’s face in order to enhance the processing of emotionally salient or threat-related stimuli (S. D. Pollak et al., 2000; Marusak et al., 2015). High stress levels could thus lead to changes in the neural system, such as alterations in brain circuitry (i.e., medial prefrontal cortex, mPFC; hippocampus; and striatal circuit) that (1) lead not only to disturbances in appraisal–reappraisal (cognitive–emotional modulation) of trauma-related stimuli but also to ordinary social and emotional stimuli (Etkin and Wager, 2007) and (2) have an effect on fear conditioning or habituation and stimulus generalization or resistance to extinction (Garfinkel et al., 2014).

Several studies have employed electroencephalography (EEG) to investigate emotional information processing by examining event-related potentials (ERPs) in patients with posttraumatic stress disorder (PTSD) (i.e., Karl et al., 2006). Two studies (Cicchetti and Curtis, 2005; Curtis and Cicchetti, 2013) considered threat-related stimuli processing specifically in maltreated children and demonstrated amplitude modulation of face-specific ERP component N170, involved in facial encoding. MacNamara et al. (2013) also confirmed the modulation of structural encoding of emotional faces [the vertex positive potentials (VPPs), corresponding to the activation in the face fusiform area (FFA)] in combat veterans with PTSD compared with non-PTSD controls. These studies both confirmed difficulties in emotion-related stimulus discrimination and in selective attention to emotional faces; these difficulties were associated with hyperarousal symptoms of PTSD, whether due to maltreatment and other interpersonal violence (IPV) or combat exposure.

High-density EEG (HD-EEG), due to its accurate temporal and spatial resolution, is an important tool to examine the early stages of brain activation during face-processing and emotion appraisal. Based on the literature, three specific ERPs, occurring early in time after visual stimuli presentation, are particularly pertinent (Berchio et al., 2017; Schwab and Schienle, 2017). The present study focused on these evoked potentials, listed below, using microstates analysis *via* cluster-maps:

(1) P100: an early visual component appearing around 100 ms after stimulus presentation, generated in the primary visual cortex and associated with posterior positive deflection, which has been described as corresponding to attentional processing of faces (Schwab and Schienle, 2017);
(2) N170: an evoked potential appearing around 170 ms after stimulus onset, which is a specific negative deflection known to reflect structural encoding of faces (Curtis and Cicchetti, 2011) and is generated in the occipito-temporal sulcus (in the fusiform gyrus);
(3) Late positive potential (LPP): appears between 200 and 500 ms after stimulus presentation and corresponds to higher levels of cognitive processing, such as the amount of attentional resources allocated to the stimulus (Perizzolo et al., 2019).

In the present study, we asked a sample of women, who had been enrolled with their children in the Geneva Early Childhood Stress Project (GECS-Pro) (Schechter et al., 2017), to respond to a validated set of male avatars during a face-evaluation task. This sample contained IPV-exposed women with related lifetime PTSD as well as non-traumatized controls. Based on previous results from this sample (Schechter et al., 2015; Perizzolo et al., 2019) and in another neuroimaging study reporting changes in emotional information processing associated with PTSD (MacNamara et al., 2013), we hypothesized that significant associations would be found among women’s lifetime diagnosis of IPV-PTSD and their evaluation of the degree of threat posed by facial avatars along dominance and trustworthiness dimensions. We hypothesized that significant group differences would be found in cortical activation between IPV-PTSD vs non-PTSD individuals.

Specifically, we expect to find:

(1) Associations between (a) under-appraisal of dominance, (b) over-appraisal of trustworthiness, and (c) IPV-PTSD diagnosis;
(2) Altered emotional processing as reflected by EEG microstates showing distinct cluster-maps in individuals with IPV-PTSD vs non-PTSD controls, the latter associated with evoked potentials P1, N170, and LPP;
(3) Altered cortical activity in individuals with IPV-PTSD vs non-PTSD controls, using source localization marked by increased activity in limbic regions and the FFA and reflecting difficulties in facial encoding represented by the N170 component; along with decreased prefrontal activity, corresponding to impairment in facial decoding (i.e., attentional processing) as observed in prefrontal regions during the LPP component.

To our knowledge, no published studies have yet described the effects of IPV-PTSD on facial processing along dominance and trustworthiness dimensions, particularly with respect to brain electrophysiology.

## Materials and Methods

### Participants

The present study obtained ethical committee approval from the Geneva University Hospitals and Faculty of Medicine in accordance with the Helsinki Declaration (World Medical Association Proposed revision of the Declaration of Helsinki, BME, 1999). We recruited a sample of women (i.e., mothers) who had participated in the GECS-Pro Phase 1. We nested the present study within the larger Phase 2 follow-up of that Phase 1 sample. By the time the present study had been funded and approved, the Phase 2 study was already half-completed. Thirty women gave informed consent to return for the additional visit needed to complete the present study. The GECS-Pro had already excluded participants who were active substance-abusers, psychotic, or physically and/or mentally impaired to preclude task participation.

### Clinical Assessment

History of experience of IPV (i.e., exposure to domestic violence, physical and/or sexual abuse, among other life events) and other traumatic events during childhood and adulthood was assessed using the Brief Physical and Sexual Abuse Questionnaire (BPSAQ; Marshall et al., 1998) and the Traumatic Life Events Questionnaire (TLEQ; Kubany et al., 2000), respectively. The BPSAQ showed strong content validity and was correlated to the TLEQ validated measure (*r* = 0.79, *p* < 0.001) (Schechter et al., 2005).

Interpersonal violence-PTSD was assessed during Phase 1 of the GECS-Pro (Schechter et al., 2017) using the Clinician Administrated PTSD Scale (CAPS) and the Posttraumatic Symptom Checklist—Short Version (PCL-S). Two groups of women were identified according to stringent criteria. The IPV-PTSD group was required to meet cut-off scores of 50 and above on the CAPS regarding lifetime symptoms and/or 40 and above on the PCL-S regarding current symptoms in the prior month, which scores were considered in light of clinical judgment based on interviews that encompassed the degree of distress and dysfunction the participant experienced. Participants could still be in the group either if the CAPS was 40 or above or if the PCL-S was 30 or above as long as the score on the other of the two PTSD measures was above the set diagnostic threshold and if the semi-structured interview determined that the participant’s distress and/or dysfunction reached a clinically significant level. Diagnostic criteria according to the Diagnostic and Statistical Manual of Mental Disorders, fourth edition, text revision (DSM-IV-TR) were applied (American Psychiatric Association, 2000).

The non-PTSD control group included women both with (36%) and without (64%) trauma exposure many with IPV. These women were only part of the non-PTSD control group only if they did not meet the criteria for PTSD diagnosis and did not have any clinically significant PTSD symptoms at a sub-threshold level. This meant that their CAPS scores had to be less than 30 and their PCL-S scores less than 25 in addition to clinician assessment by interview.

Participants’ depressive symptoms (using the Beck Depression Inventory II—BDI-II; Beck and Steer, 1987) were also assessed during Phase 1 since major depressive disorder (MDD) is often comorbid with PTSD in as many as 40–50% of cases (Flory and Yehuda, 2015). We controlled for history of lifetime depression in our analyses.

Family socio-economic status (SES) was calculated using the Largo Index (Largo et al., 1989) using the Geneva Sociodemographic Questionnaire (GSQ) (Sancho Rossignol et al., unpublished). The two groups were age- and laterality-matched based on data from Phases 1 (2010–2014) and 2 (2014–2018) of the GECS-Pro. Demographic and clinical characteristics are presented in Table 1.

**TABLE 1:** Demographic and clinical characteristics of IPV-PTSD and non-PTSD participants.

Participant variables	All	Controls (*n* = 14)	IPV-PTSD (*n* = 16)	*p*-value
Age of participants (in years)	38.31 (6.99)	37.67 (7.76)	39.00 (6.29)	0.617
Socio-economic status (higher score means lower status)^1^	4.96 (1.97)	4.42 (1.98)	5.43 (1.91)	0.197
% Left-handed	0% (0/30)	0%	0%	1
% History of prior drug and alcohol use^1^	6.7% (2/30)	6.7% (1/15)	6.7% (1/15)	1
Depression (BDI)^1^	9.41 (8.18)	4.42 (3.34)	13.40 (8.78)	0.002*
CAPS total score^1^	53.48 (31.92)	23.50 (9.63)	77.47 (20.79)	0.000***
Dissociation symptoms^1^	5.88 (6.83)	2.80 (4.66)	8.07 (7.42)	0.044*

% (*n*/number of valid data) and mean (SD) are reported. ^1^There were three missing values within the control group. *p < 0.05. ***p < 0.001.

Electroencephalography was recorded during a single session, during which clinicians also interviewed participants and updated their life events histories that they may have experienced since Phase 1 of the GECS-Pro. Participants filled the PCL-S out again in order to evaluate their current PTSD level at the time of EEG recording.

In our final sample, 16 women met the criteria for lifetime DSM-IV-TR PTSD diagnosis (mean age = 39.00 years, SD = 6.29), and 14 women (mean age = 37.67 years, SD = 7.76) formed the non-PTSD control group. Among the non-PTSD control group, five women were exposed to violent traumatic events. Behavioral data and HD-EEG were collected for all 30 participants. Finally, 29 out of 30 participants’ data were viable for analysis. No participants showed neurological abnormalities.

### Behavioral Task

Participants evaluated 500 avatars that varied along two dimensions of dominance and trustworthiness. These avatars were created using the Facegen Modeller program^1^ based on a data-driven computational model that visualizes perceptions of trustworthiness and dominance^2^. The avatar database was validated and demonstrated strong interrater reliability (Oosterhof and Todorov, 2008; Todorov et al., 2013). Each avatar presented sits along a continuum from “non-dominant” (or “Dominance = 1”) and “untrustworthy” (or Trust = 1) to “dominant” (“Dominance = 5”) and “trustworthy” (“Trust = 5”), with “neutral” (“Dominance = 3” and “Trust = 3”) as a middle reference.

**FIGURE 1:**
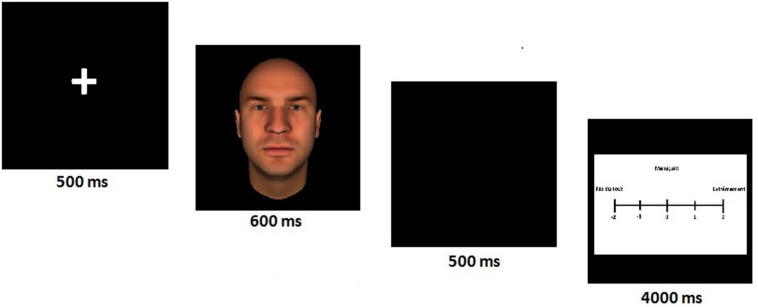
The evaluation task of avatars’ faces. The task required participants to attribute a value as to how dominant/trustworthy the avatar was, on a scale from -2 to + 2. This paradigm was adapted for EEG.

This paradigm was adapted for recording ERPs. We presented avatar faces in the center of the screen (22.5 cm × 21 cm). The latter was then followed by the participant’s being asked to evaluate the avatar on a scale from -2 to +2. Using this scale, women had to attribute a value as to how dominant, or in other words, dominant and trustworthy the index avatar was. Avatars were presented for 600 ms on a black background, preceded by a fixation cross (randomly varied between 905 and 1,135 ms); then participants were asked to evaluate the stimulus noted above (4 s) (Figure 1).

Visual stimuli were distributed into four blocks, with an alternation of blocks presenting trustworthiness-related avatars only and blocks displaying dominant-related avatars only. Each block was composed of 125 stimuli (25 different pseudo-randomized avatars in five pseudo-randomized gradations as described above). Some necessary breaks during the EEG recording were scheduled so that participants could get some rest and stay focused during the entire task. Participants evaluated avatars using a multifunctional response console (chrono; PSYCHOLOGY SOFTWARE TOOLS, INC.).

### EEG Data Acquisition and Pre-processing

Electroencephalography data were acquired using a 256-channel Electrical Geodesic Inc. System (Eugene, OR, United States) at a sampling rate of 1,000 Hz and with Cz as the acquisition reference. Electrode impedances were kept below 30 kohm, and offline analyses were performed using Cartool 3.60 (4698) software^3^. EEG epochs were segmented 100 ms before and 600 ms after stimulus onsets and were digitally filtered offline at 0.1–40 Hz (causal filter, 24 db/octave roll-off). A notch-filter was also applied. We excluded electrodes that had been placed on the neck and jaw, leaving 204 electrodes analyzed. EEG data were visually inspected during averaging and processing. Epochs containing artifacts were excluded from further analysis. Artifactual electrodes were then interpolated using spherical spline interpolation methods (Perrin et al., 1989). ERP data were recalculated against the average reference.

### Analysis of Behavioral Data

After exploratory analysis using box and scatter plots to detect outliers for each analysis, repeated measures ANOVA was used to compare the evaluation of the avatars with dimensions (i.e., dominance and trustworthiness) as within-subject factors and group (IPV-PTSD vs non-PTSD groups) as a between-subject factor. Continuous analyses were then performed using Spearman’s rank correlation coefficients. We controlled for current depression by entering any significant associations into a multiple linear regression model. Because a number of our behavioral measures were not normally distributed and/or could not be assumed to be at interval rather than ordinal scale, using non-linear analysis seemed inappropriate, and non-parametric linear analysis was used for correlations instead. Group differences were tested parametrically where they referred to interval scaled EEG results. Statistics were computed using SPSS version 22 (IBM Corporation, Armonk, NY, United States). Alpha levels were set to *p* < 0.05 for all tests; Bonferroni corrections were applied for all comparisons.

### ERP Analyses

We analyzed women’s brain activity in response to avatar-presentation using global scalp analysis and EEG source imaging methods (Murray et al., 2008, 2009; Berchio et al., 2017).

#### Segmentation Into Microstates

For each dimension, the 10 grand mean ERPs were jointly submitted to a k-means cluster analysis (Brunet et al., 2011), which is a classical repeated-pattern-of-recognition method, resulting in a certain number of prototype-maps (or cluster-maps) that best fit the whole dataset. An optimal-number-of-clusters count was obtained using the Krzanowski–Lai (KL) criterion, which is determined by the L-corner of the dispersion curve. This is an accurate clustering method that is set when an additional cluster does not lead to a significant gain of the global quality (Brunet et al., 2011). The analysis was run using Cartool software, and the cluster analysis was completely data-driven, thus blinded to condition and map group-assignment. We requested 300 random trials on 10 ERPs of 600 ms duration at 250 Hz for dominance and trustworthiness dimensions.

The resulting cluster-maps were then back-fitted to the grand mean data by assigning each individual map to the cluster-map on two different fitting variables [i.e., number of time-frames and global explained variance (GEV)]. The number of time-frames allowed us to determine how long a given cluster-map was present during specific time-intervals (N170 and LPP evoked potentials). Back-fitting of cluster-maps for GEV helped us to consider how well a specific map, which had been identified in the cluster analysis, explained the dataset of each subject, in terms of both strength and frequency of occurrence of a given map (Britz et al., 2014). Individual sample *t*-tests were run on each parameter.

### ERP Source Analyses

We performed analyses in the source space using a Local Auto Regressive Average (LAURA) inverse solution model (de Peralta Menendez et al., 2001; Michel et al., 2001). Current density distribution was calculated for 5,018 solution points located in the gray matter of the adult template head model (MNI brain^4^). The local spherical model with anatomical constraints (LSMAC) was used for the forward-solution (Brunet et al., 2011; Birot et al., 2014). The current density values were averaged across the N170 (144–200 ms) and LPP (192–388 ms—dominance dimension, 192–444 ms—trustworthiness dimension) components time window and then subjected to randomization test (i.e., 5,000 permutations, *p*-values less than 0.05). Permutation statistics were used to adjust for multiple comparisons (Maris and Oostenveld, 2007). *t*-Values were extracted in order to know the direction of effect.

## Results

**FIGURE 2:**
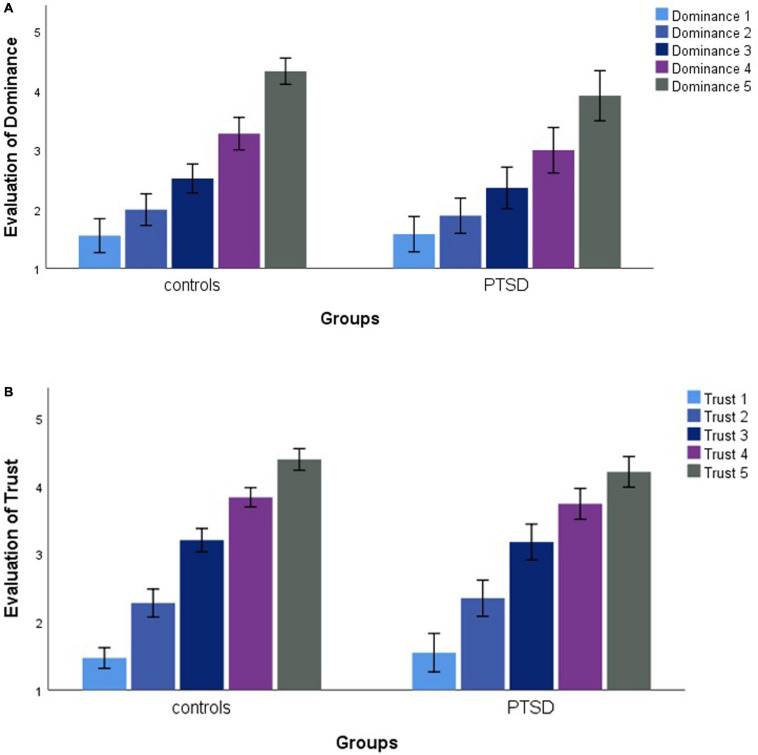
Mean evaluation of avatars regarding dominance (Dominance = 1–5) **(A)** and trustworthiness (Trust = 1–5) **(B)** for each group (IPV-PTSD vs non-PTSD controls).

With respect to our hypothesis, results could be summarized as follows:

1) IPV-PTSD lifetime severity was associated with decreased appraisal of dominance (Dominance = 5; *r* = -0.486, *p* = 0.010, *N* = 27) but not with higher appraisal for trustworthiness. A similar relationship in dominance could, however, not be found for IPV-PTSD diagnosis alone. For more details, see Figures 2A,B and Table 2 and Section “Behavioral Results.”

**TABLE 2:** Spearman’s correlations between participants psychopathology, exposure to violent events, and avatar’s evaluation.

Spearman’s correlations		Non-dominant (Domin = 1)	Relatively non-dominant (Domin = 2)	Neutral dominance (Domin = 3)	Relatively dominant (Domin = 4)	Very dominant (Domin = 5)	Non-trustworthy (Trust = 1)	Relatively non-trustworthy (Trust = 2)	Neutral trustworthy (Trust = 3)	Relatively trustworthy (Trust = 4)	Very trustworthy (Trust = 5)
Lifetime PTSD: CAPS total score	r	0.085	−0.061	−0.143	−0.202	−0.278	−0.018	0.17	−0.005	−0.154	−0.31
	*p*	0.673	0.763	0.475	0.312	0.16	0.928	0.396	0.982	0.443	0.116
	*N*	27	27	27	27	27	27	27	27	27	27
Actual PTSD: PCLS total score	*r*	0.19	0.216	0.094	−0.16	−0.282	0.126	0.337	0.156	−0.155	−0.234
	*p*	0.323	0.261	0.628	0.408	0.138	0.516	0.074	0.418	0.422	0.221
	*N*	29	29	29	29	29	29	29	29	29	29
Re−experiencing symptoms	*r*	0.073	−0.068	−0.196	−0.235	−0.297	0.011	0.208	0.113	0.02	−0.186
	*p*	0.716	0.735	0.327	0.237	0.133	0.955	0.299	0.575	0.923	0.352
	*N*	27	27	27	27	27	27	27	27	27	27
Avoidance symptoms	*r*	−0.88	−0.035	−0.097	−0.143	−0.165	−0.054	0.084	−0.123	−0.251	−0.311
	*p*	0.663	0.863	0.632	0.476	0.41	0.79	0.677	0.54	0.206	0.115
	*N*	27	27	27	27	27	27	27	27	27	27
Hyperarousal symptoms	*r*	0.053	−0.059	−0.201	−0.363	−0.486	0.12	0.367	0.105	−0.21	−0.347
	*p*	0.793	0.768	0.316	0.063	0.01	0.552	0.059	0.603	0.293	0.076
	*N*	27	27	27	27	27	27	27	27	27	27
Dissociation symptoms	*r*	0.115	0.141	−0.013	−0.103	−0.149	−0.002	0.345	0.366	0.351	0.079
	*p*	0.593	0.512	0.951	0.631	0.486	0.992	0.099	0.079	0.092	0.715
	*N*	24	24	24	24	24	24	24	24	24	24
Witnessed DV as a child	*r*	0.223	0.169	−0.007	−0.118	−0.202	0.227	0.474	0.386*	0.064	−0.001
	*p*	0.264	0.401	0.972	0.557	0.313	0.254	0.013	0.047*	0.752	0.996
	*N*	27	27	27	27	27	27	27	27	27	27
TLEQ N of violent events	*r*	0.173	0.053	−0.136	−0.321	−0.526	0.133	0.358	0.088	−0.084	−0.289
	*p*	0.388	0.794	0.498	0.102	0.005	0.509	0.067	0.664	0.676	0.144
	*N*	27	27	27	27	27	27	27	27	27	27

**FIGURE 3:**
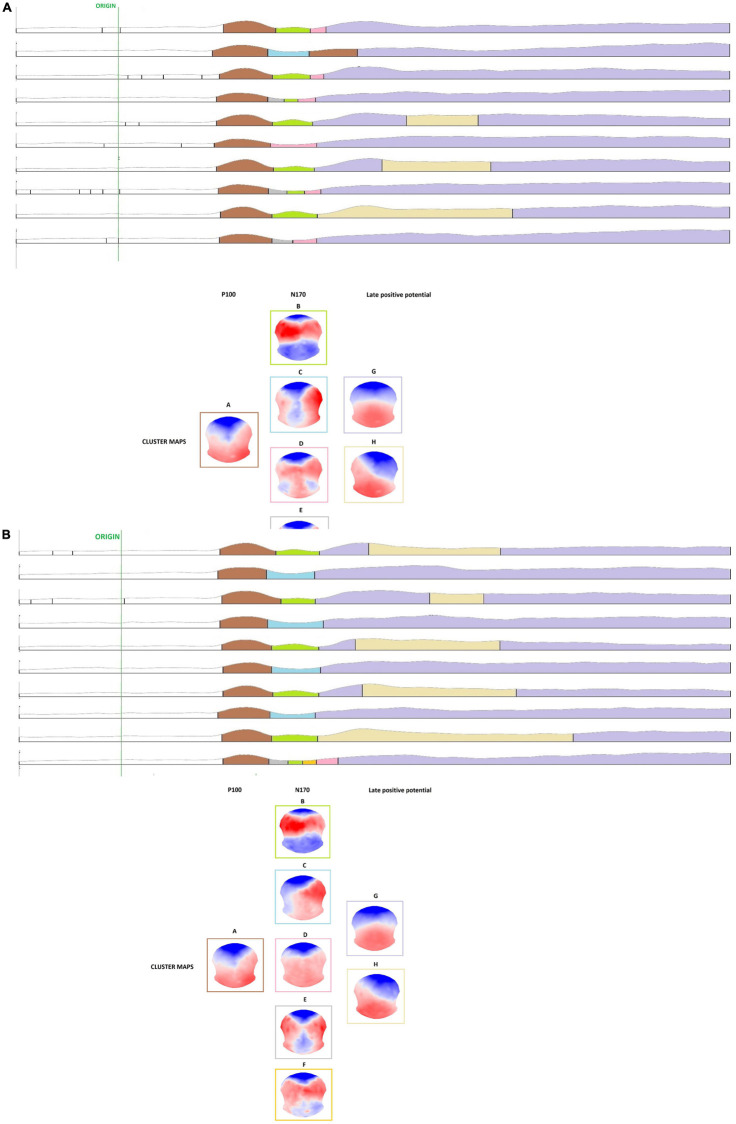
Cluster analysis of the grand mean ERPs where microstates are presented in different colors, as well as their corresponding cluster-maps. **(A)** Microstates of the five degrees of the dominance dimension (Dominance = 1–5) and for each group, as well as corresponding topographical maps. Each evoked potential was represented by specific microstates and topographical maps (P1 = microstate Class A; N170 = microstate Classes B, C, D, E, and F; LPP = microstate Classes G and H). **(B)** Microstates of the five degrees of the trustworthiness dimension (Trust = 1–5) and for each group, as well as corresponding topographical maps. Each evoked potential was represented by specific microstates and topographical maps (P1 = microstate Class A; N170 = microstate Classes B, C, D, and E; LPP = microstate Classes G and H).

**FIGURE 4:**
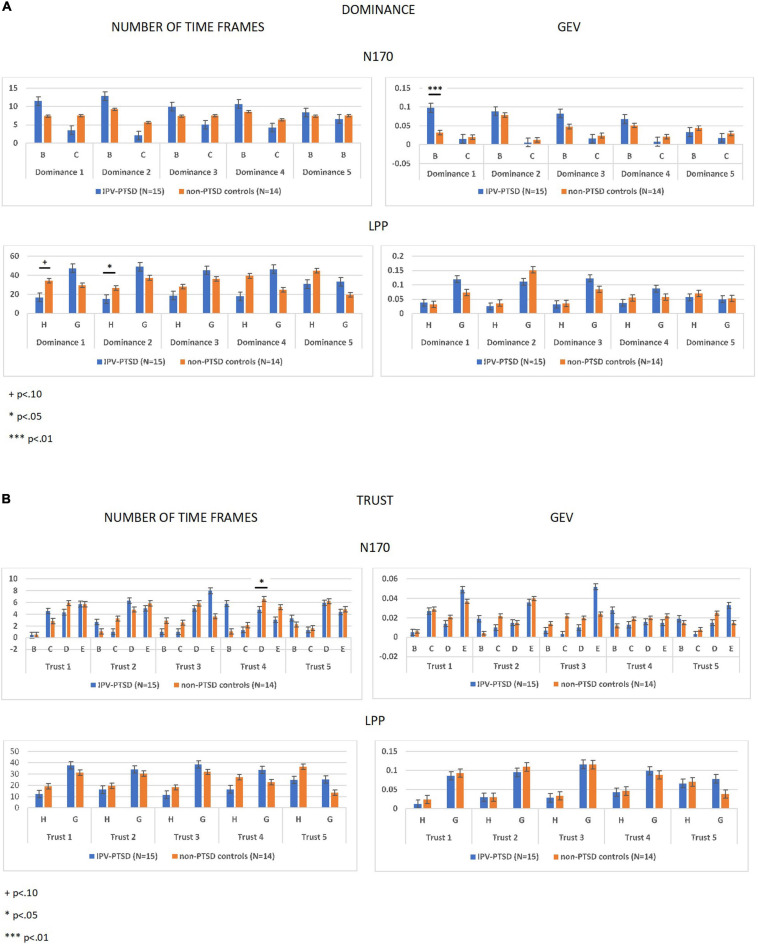
Fitting statistics using independent sample t-tests on number of time-frames and global explained variance (GEV) parameters. Fitting statistics were run on each degree of dominance (Dominance = 1–5) **(A)** and trustworthiness (Trust = 1–5) **(B)** dimensions and regarding each group (IPV-PTSD vs non-PTSD controls).

*Not significant with FDR correction for multiple comparisons.
2) Cluster-maps differed between IPV-PTSD and non-PTSD control groups with respect to evoked potentials N170 and LPP but not P1 for both dominant and trustworthy avatars. For more details, see Figures 3A,B,4A,B and Section “ERP Segmentation in Microstates.”

**FIGURE 5:**
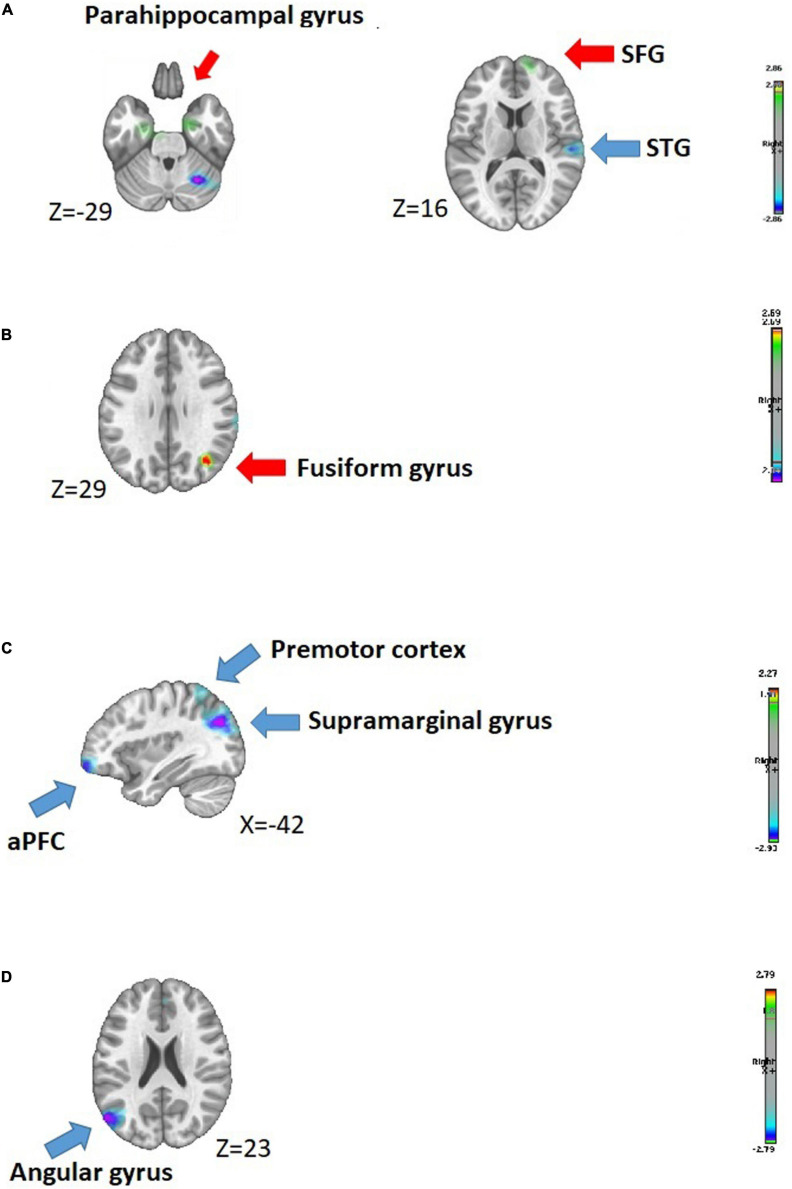
Source localizations analysis. **(A,B)** Inverse solutions conducted for non-dominant (Dominance = 1) avatars, measured in evoked potentials N170 and LPP, respectively. **(C,D)** Inverse solutions conducted for relatively trustworthy (Trust = 4) avatars, measured in evoked potentials N170 and LPP, respectively.

In response to “non-dominant” avatars (Dominance = 1) within the N170 component, we noted that the IPV-PTSD group showed increased activity in limbic regions, compared with non-PTSD controls. In the LPP component in response to “non-dominant” avatars, individuals with lifetime IPV-PTSD, as compared with non-PTSD controls, displayed increased right activation in fusiform gyrus. Additionally, for both the N170 and LPP, the IPV-PTSD group displayed decreased left activity in the anterior prefrontal cortex (aPFC), compared with non-PTSD controls response to “relatively trustworthy” (Trust = 4). For more details, see Table 3 and Figure 5 and Section “Inverse Solution.”

**TABLE 3:** EEG source analysis results.

	Location	Lateralization	MNI			BA	*p*-Values	*t*-values
			*x*	*y*	*z*			
Non-dominant avatars (Dominance = 1)								
N170	Parahippocampal gyrus	R	23	−3	−36	36	0.042	2.13
	Ventral entorhinal cortex	L	16	−3	−29	28	0.048	2.07
	Medial frontal gyrus	R	16	62	10	10	0.046	2.09
	Superior frontal gyrus	R	16	68	16	10	0.047	2.08
	Inferior temporal gyrus	R	29	−3	−42	20	0.05	2.05
	Superior temporal gyrus	R	68	−29	16	22	0.034	–2.23
	Supramarginal gyrus	R	62	−23	16	40	0.015	–2.59
	Visuo−associative cortex	L	−10	−74	−3	18	0.02	2.46
	Cerebellum	R	29	−62	−36		0.015	–2.6
LPP	Fusiform gyrus	R	42	−62	10	37	0.018	2.51
	Angular gyrus	R	36	−68	29	39	0.012	2.69
	Associative visual cortex	R	42	−62	16	19	0.02	2.48
Relatively trustworthy avatars (Trust = 4)								
N170	Anterior PFC	L	−42	55	10	10	0.047	–2.08
	Supramarginal gyrus	L	−62	−55	29	40	0.048	–2.07
	Premotor cortex	L	−16	−10	68	6	0.044	–2.11
	Cerebellum	R	23	−62	−29		0.047	–2.08
LPP	Visuo−motor coordination	R	3	−55	55	7	0.025	–2.37
	Angular gyrus	L	−42	−74	29	39	0.02	–2.46

### Behavioral Results

We first considered whether the participants’ evaluation of the facial avatars along dimensions of dominance and trustworthiness would significantly differ between groups (IPV-PTSD vs non-PTSD controls). Results of this repeated measures ANOVA showed a significant main effect of the dominance dimension (*F* = 237.03, *p* = 0.000, *N* = 30), but no main effect by group (*F* = 1.122, *p* = 0.299, *N* = 30), nor by group × dimension interaction (*F* = 1.646, *p* = 0.210, *N* = 30). Results of the repeated measures ANOVA to analyze participants’ evaluation of the avatars along the trustworthiness dimension also demonstrated a significant main effect of the trustworthiness dimension (*F* = 494.99, *p* = 0.000, *N* = 30) but no main effect by group (*F* = 0.085, *p* = 0.773, *N* = 30) nor by group × dimension interaction (*F* = 1.176, *p* = 0.287, *N* = 30). Results are displayed in Figures 2A,B.

Continuous analyses using Spearman’s correlations were then performed to examine associations between participants’ evaluation of the facial avatars along the dominance and trustworthiness dimensions and the following three sets of measures:

1) Lifetime IPV-PTSD overall, subscale symptom severity (i.e., thus providing more statistical power than analysis by categorical diagnosis) and current IPV-PTSD overall and subscale symptom severity at the time of EEG recording. Results demonstrated that participants’ lifetime PTSD hyperarousal symptom cluster severity significantly correlated with their appraisal of “dominant” avatars (Dominance = 5; *r* = −0.486, *p* = 0.010, *N* = 27). This result survived the Bonferroni correction (*p* = 0.040). However, the PTSD re-experiencing and avoidance symptom clusters showed no significant associations. Current IPV-PTSD overall and subscale symptom severity at the time of EEG recording similarly was not significantly associated with participants’ evaluation of the avatars along the dimensions of dominance or trustworthiness (all *r*s < 0.337, all *p*s > 0.074, *N* = 27).

Multiple linear regression was used to control for lifetime depressive symptoms while examining if IPV-PTSD hyperarousal symptoms would remain a predictor of women’s evaluation of the degree of dominance perceived among the avatars. Lifetime depressive symptoms did not significantly alter the model even though PTSD hyperarousal subscale symptoms overlapped with the depressive symptoms (i.e., disturbance of sleep, poor concentration, irritability) (all *F* = 3.095, *p* = 0.064).

2) Related lifetime dissociative symptom severity. Lifetime dissociative symptom severity was not significantly associated with the evaluation of the degree of dominance or trustworthiness shown by the facial avatars (*p* < 0.13).

Associations between IPV-PTSD symptom severity and evaluation of avatars are summarized in Table 2.

3) We then applied Spearman’s correlations in order to examine possible associations between the number of violent events participants experienced over their lifetime with participants’ evaluation of facial avatars along the dimension of dominance and trustworthiness. Evaluating the facial avatars as being less dominant was significantly associated with a higher number of lifetime violent events experienced (Dominance = 5; *r* = −0.526, *p* = 0.005, *N* = 27). This result survived Bonferroni correction (*p* = 0.015). None of the other comparisons between individuals’ exposure to violence and evaluation of the facial avatars along the dominance dimension reached significance. Considering more specifically women’s exposure to domestic violence as a child, we found a significant association with over-evaluation of trust in “relatively untrustworthy” avatars (Trust = 2; *r* = 0.474, *p* = 0.013, *N* = 27) and with “neutrally trustworthy/untrustworthy” avatars (Trust = 3; *r* = 0.386, *p* = 0.047, *N* = 27). After application of the Bonferroni correction, the association between evaluation of “relatively untrustworthy” avatars and individuals’ exposure to domestic violence as a child remained significant (*p* = 0.039), whereas the other associations did not.

**FIGURE 6:**
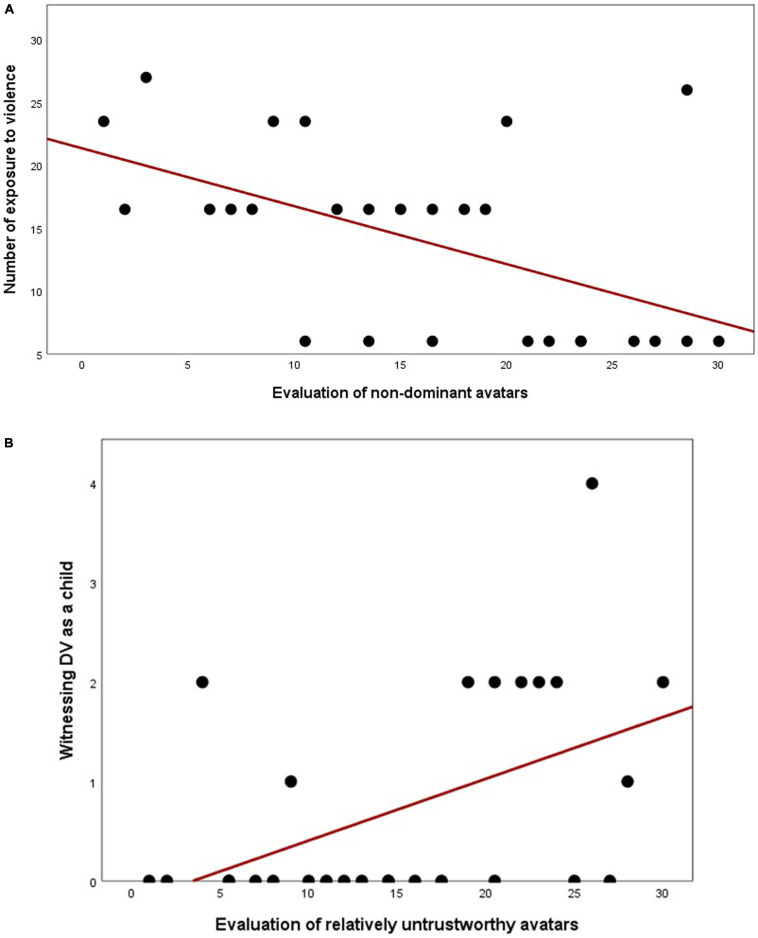
Scatter plots rank-based Spearman’s correlations between participants’ evaluation of non-dominant avatars and exposure to violent events **(A)** and between evaluation of relatively untrustworthy avatars and witnessing domestic violence as a child **(B)**.

Associations between exposure to violence and evaluation of avatars are summarized in Table 2 and, scatter plots based on ranks are presented in Figure 6.

### EEG Results

#### ERP Segmentation in Microstates

We first processed cluster analyses on the grand mean ERPs for both dominance- and trustworthiness-related avatars. Each main evoked potential (P1, N170, and LPP) elicited during the 600 ms post-stimulus presentation is visible for the control group. This replicates findings obtained in previous EEG-related face processing studies (Berchio et al., 2017; Schwab and Schienle, 2017). In the PTSD-group, evoked potentials P1 and LPP were visible and comparable to the literature, whereas N170 component did not appear on grand averaging in response to both dominance- and trustworthiness-related avatars. Regarding amplitude and latency of the main evoked potentials, P1 and LPP looked very similar in response to each of the five gradations along both the dominance and trustworthiness dimensions and within both groups.

Then, cross-validation analyses using the KL criterion showed that nine cluster-maps best explained the whole 300 maps entered in the cluster analysis and did so for both dominance and trustworthiness conditions.

##### Microstates in dominance-related avatars

Temporal segmentation analysis allowed us to identify periods of stable electric field topographies (i.e., microstates) that were linked to evoked potentials and elicited in response to avatar-presentation. In response to dominance-related avatars, we first noted that a microstate Class “A” was observed during the P1 component, for both groups. However, cluster-maps then differed between the IPV-PTSD and non-PTSD control groups with respect to evoked potentials N170 and LPP.

Cluster-maps elicited in the N170 component, corresponding to face-encoding, revealed group differences in the IPV-PTSD vs control group displaying microstate Class “C.” The former was characterized by a specific pattern of left-lateralized negativity and right-lateralized positivity. The control group showed a microstate Class “B” and was characterized by typical occipital negativity that is usually reflected in the N170 component.

Finally, cluster-maps elicited in the LPP evoked potential, which corresponds typically to face decoding, also revealed group differences: the IPV-PTSD group displayed microstate Class “G” only, with reduced occipital positivity, whereas the control group displayed a sequence of microstate Classes “G” and “H,” with increased occipital positivity.

##### Microstates in trustworthiness-related avatars

In response to trust-related avatars, and as previously observed along the dominance dimension, microstate Class “A” was measured in P1 component, for both groups (see Figure 3B). Cluster-maps differed again between IPV-PTSD and control groups for evoked potentials N170 and LPP.

Cluster-maps elicited in the N170 component revealed group differences in the IPV-PTSD vs control group displaying microstate Classes “C,” “D,” and “E” or a succession of these maps, whereas the control group showed a microstate Class “B,” characterized by typical occipital negativity that is usually reflected in the N170 component.

Finally, cluster-maps elicited in the LPP evoked potential also revealed group differences: the IPV-PTSD group displayed again microstate Class “G” only, with reduced occipital positivity, whereas the control group displayed a sequence of microstate Classes “G” and “H,” with increased occipital positivity.

#### Fitting Statistics of the ERP Topographies

Segmentation in microstates led us to test hypotheses regarding topographic modulation at the group level, using a statistical fitting procedure. We fitted each cluster-map to the individual evoked potentials of each subject for N170 and LPP evoked potentials, in which we previously observed group differences in microstate segmentation. We considered two parameters, namely, (1) the number of time-frames (duration) and (2) the GEV for each cluster-map.

##### Fitting statistics in response to dominance-related avatars

Considering topographical maps corresponding to the N170 component, in response to dominance-related avatars, we ran independent sample *t*-test on microstate Classes “B” and “C.” Results demonstrated significant between-group differences in “GEV” parameter in response to “non-dominant” avatars (Dominance = 1; *t* = -3.306, *p* = 0.003, *N* = 29). The number of time-frames did not differ between groups.

We then considered topographical maps corresponding to the LPP component in response to dominance-related avatars and ran independent sample *t*-test on microstate Classes “G” and “H” in order to investigate between-group differences. We noted significant between-group differences in response to “relatively dominant avatars (Dominance = 4)” with respect to the number of time-frames (i.e., measured in cluster-map G: *t* = 2.267, *p* = 0.032, *N* = 29 and cluster-map H: *t* = -2.267, *p* = 0.032, *N* = 29, respectively). Detailed results are presented in Figure 4A.

##### Fitting statistics in response to trustworthiness-related avatars

Considering topographical maps corresponding to the N170 component, in response to trustworthiness-related avatars, we ran independent sample *t*-test on microstate Classes “B,” “C,” “D,” and “E,” in order to investigate group differences. Results demonstrated significant between-group differences in the number of time-frames parameter, in response to “relatively trustworthy” avatars (Trust = 4; *t* = -2.464, *p* = 0.021, *N* = 29), whereas GEV parameter did not show any group differences.

We considered topographical maps corresponding to the LPP component in response to trustworthy-related avatars by running fitting statistics on microstate Classes “G” and “H” and found no significant group differences. Detailed results are presented in Figure 4B.

Fitting statistics indicated that non-dominant and relatively trustworthy avatars had the best fit. We continued the analysis by running the source localization exclusively on these conditions, along these dimensions, for both N170 and LPP evoked potentials.

#### Inverse Solution

##### Source localization in dominance-related avatars

When considering between-group differences in brain activity in response to “non-dominant” avatars (Dominance = 1) within the N170 component, we noted that the IPV-PTSD group showed increased activity in limbic regions, namely, within the right parahippocampal gyrus and the left ventral-entorhinal cortex (EC), compared with non-PTSD controls. Individuals with IPV-PTSD also demonstrated increased right activation in the middle frontal gyrus (MFG) and superior frontal gyrus (SFG), as well as increased right activity in inferior temporal gyrus but decreased right activation in the superior temporal and supramarginal gyri, during the N170 component, as compared with controls. Finally, we observed between-group differences measured as characterized by increased activity in the left visuo-associative cortex and decreased activation in the right cerebellum.

With regard to brain activation in the LPP component in response to “non-dominant” avatars, individuals with lifetime IPV-PTSD, as compared with non-PTSD controls, displayed increased right activation in fusiform and angular gyri. The IPV-PTSD vs control group also demonstrated increased right activity in the associative visual cortex.

##### Source localization in trustworthiness-related avatars

We considered between-group differences in brain activity in response to “relatively trustworthy” (Trust = 4) avatars in relation to both N170 and LPP evoked potentials. Regarding the face-encoding-related component, individuals with IPV-PTSD displayed decreased left activity in the aPFC and in the supramarginal gyrus, compared with non-PTSD controls. IPV-PTSD participants vs controls also demonstrated decreased left activity in the premotor cortex as well as decreased activation in the right cerebellum.

In response to “relatively trustworthy” avatars, brain activity measured in the face-decoding-related component showed between-group differences with the IPV-PTSD group displaying comparatively decreased activation in the right visuo-motor coordination cortex and in the left angular gyrus.

Table 3 and Figure 5 display the detailed results regarding to source localization.

## Discussion

In the present study, we considered how lifetime diagnosis of IPV-PTSD might modulate the appraisal of threat in response to facial avatars along component dimensions of dominance and trustworthiness. Both behavioral and EEG findings confirmed that women with IPV-PTSD presented a bias in face processing (encoding and decoding) along with greater difficulty in evaluating dominance and trustworthiness when attributing social judgment of facial avatars in the assigned task.

### Discussion of Results Relating to Hypothesis 1

We found a significant association between psychopathology and exposure to violence and under-estimation of dominance and/or over-estimation of trustworthiness confirming this first hypothesis. Evaluation of dominant avatars as less dominant was associated with the number of lifetime violent events to which the women had been exposed and related lifetime PTSD diagnosis, and in particular, the severity of hyperarousal cluster symptoms. The literature has demonstrated that hypervigilance or dysfunctional threat detection associated with PTSD was explained by the development of preferential attention to threatening stimuli, leading to exaggerated reactivity in afflicted individuals, in a goal of adapting to their environment and thus survival (Darwin, 1965; MacNamara et al., 2013; Shalev et al., 2017). The present study has added that, beyond the diagnosis of PTSD, severity of IPV exposure as defined by the number of exposures to violent events modulates appraisal of threat.

Most of the IPV-PTSD group in the present study had also experienced abuse and/or witnessed domestic violence as part of their family life during childhood. Reduced recognition of risk in interpersonal male–female behavior has been linked to prior experience of physical and sexual violence including victimization during puberty (Wilson et al., 1999; Bockers et al., 2014). One could therefore speculate that dominant men may seem more familiar to the IPV-PTSD group. These women may have thus habituated to more dominant men given that these men might have more likely been among their attachment figures, which in turn may influence how they rate trust and dominance. Under-appraisal of dominance and over-appraisal of trustworthiness observed in women with IPV-PTSD, compared with non-PTSD controls, may be an important finding for understanding what may underlie repeated choice of violent partners despite prior experience of victimization (R. A. Pollak, 2004; Rakovec-Felser, 2014). This latter hypothesis may also be linked to previous findings that have associated attachment anxiety in victimized women with reduced risk assessment (Bockers et al., 2014).

We also question if because of prior exposure to domestic violence and maltreatment, women may behave more submissively, as a survival tactic, and may paradoxically project their own submissiveness and vulnerability, as a form of psychological defense, onto potential male partners. One paper posits that acknowledgment of male dominance, which is related to the observation that biological evolution favors coupling with dominant male partners, is too anxiety-provoking for those women exposed to IPV (Richards et al., 1991).

Furthermore, we can wonder if in the wake of violent trauma, the cognitive vs affective and physiologic (i.e., gut reaction) components of facial appraisal may be further disassociated, particularly if the facial stimulus is less overtly expressive of affect, such as anger or threatening intention. The intensity of fear may thus be suppressed by some individuals when making a “snap-judgment” (Schachter and Singer, 1962; Davis et al., 2009). Moreover, studies have supported a more generalized psychophysiological difference in the autonomic nervous system’s processing of stressors between the sexes, supporting the “tend and befriend” hypothesis as a coping strategy that is more characteristic of females (Taylor et al., 2000; Adjei et al., 2018). This latter evolutionarily based hypothesis that may be dependent on oxytocin and endogenous opioid receptor differences posits that affiliation possibly with more dominant males (vs submission) is both a way of seeking protection and survival-enhancing resources, as well as contributing to physical healing and psychological recovery following aggression (Taylor et al., 2000; Detillion et al., 2004). One may in light of this intriguing “tend and befriend” hypothesis consider that the dominant male who turns against his own female mate may be somewhat of a Trojan horse that takes advantage of this innate coping system, thus requiring an observing third to help that female gain awareness of how her psychobiological defenses may be blinding her to risk to her survival and that of her offspring.

### Discussion of Results Relating to Hypothesis 2

Microstate results indicated that lifetime IPV-PTSD modulated emotional appraisal, both during encoding and decoding processing as measured in N170 and LPP evoked potentials. Considering the face-processing specificity of the N170 component (Hajcak et al., 2012), we note that non-PTSD controls elicited a map-dominance (cluster-map “B”) comparable to classical N170 topographical map found in the literature (Bentin et al., 1996), whereas IPV-PTSD individuals showed a predominance of a specific map (cluster-map “C”) with a pattern of left-lateralized negativity and right-lateralized positivity, in response to both dominance- and trustworthiness-related avatars, indicating a functional change in how threat-related faces are processed.

Modulation of the N170 evoked potential was commonly seen in the literature regarding individuals with PTSD (MacNamara et al., 2013) and anxiety disorders in general (Felmingham et al., 2016), but this is also the case with other psychiatric disorders (i.e., in schizophrenia; Shah et al., 2018) as well as in maltreated children (Cicchetti and Curtis, 2005; Curtis and Cicchetti, 2013).

Considering face-decoding processing measured LPP evoked potentials, our results suggested that women with PTSD always demonstrated the same microstate Class “G” across both dominance and trustworthiness dimensions, whereas non-PTSD controls usually demonstrated the implementation of microstate Class “H” in addition to cluster-map “G” observed in the LPP component. One might assume that IPV-PTSD individuals would miss or skip a processing step, corresponding to microstate Class H, in decoding the degree of dominance and trustworthiness in avatars when compared with non-PTSD controls. As timing of appearance of microstate Class “H” depended on conditions in non-PTSD controls, one may also think that both N170 and LPP evoked potentials seem less stable in women with IPV-PTSD as compared with controls. This could indicate that emotional processing is generally dysregulated, not just regionally but also temporally among traumatized women.

The present study is, however, the first to our knowledge to employ microstate analysis for both face encoding and decoding-related evoked potentials in individuals with PTSD and, thus, extends the literature, since very few clinical studies so far used segmentation in cluster-maps in their EEG analyses.

### Discussion of Results Relating to Hypothesis 3

Source localization findings corresponding to the encoding of non-dominant avatars (Dominance = 1) (measured in the N170 component) confirmed limbic dysregulation commonly observed in individuals with lifetime IPV-PTSD. Specifically, increased activity among IPV-PTSD vs non-PTSD women was measured in the right parahippocampal gyrus and in the left ventral-EC. The parahippocampal gyrus, which surrounds the hippocampus, is involved in associative learning and in memory encoding and retrieval (Werner et al., 2009). Increased activation of the right parahippocampal gyrus is commonly observed in individuals with PTSD (Werner et al., 2009; Herz et al., 2016) or in response to trauma-related stimuli (Liberzon et al., 1999) and is often associated with PTSD symptoms (Yehuda and LeDoux, 2007; Brown et al., 2016). We also noted hyper-activation in a neighboring structure: the EC, specifically thought to be implicated in contextual fear conditioning (Majchrzak et al., 2006; Sparta et al., 2014). Findings obtained in the limbic system demonstrated that, even in absence of threat, individuals with PTSD continue to maintain hypervigilance and fail to contextualize non-dominant avatars. Dysregulation of the limbic system is commonly associated with top-down regulation deficit (Garfinkel and Liberzon, 2009) that we could expect to find in our sample. However, we did not find this to be the case and only demonstrated increased activation of the right MFG and right SFG in women with IPV-PTSD compared with non-PTSD controls. One might explain these negative findings concerning top-down regulation of the limbic system by the fact that non-PTSD controls did not appear to present a specific pattern of emotion regulation when compared with the PTSD group.

Between-group differences in brain activation measured in the LPP component, in response to non-dominant avatars, showed significantly increased activity in the right fusiform and in the right angular gyri in IPV-PTSD individuals compared with non-PTSD controls. Increased activity measured in the fusiform gyrus, which contains the FFA involved in face processing and recognition (Kanwisher et al., 1997; Haxby et al., 2000), confirmed increased brain resources recruitment in processing non-dominant faces in women with IPV-PTSD compared with non-PTSD controls.

Source localization findings corresponding to the encoding of relatively trustworthy avatars (Trust = 4) (measured in the N170 component) demonstrated that individuals with IPV-PTSD presented decreased activation in the left aPFC and in the left supramarginal gyrus, in comparison with non-PTSD controls. The aPFC is involved in the allocation of attention (Ramnani and Owen, 2004), but its activation might also be modulated by the reliability of confidence judgments (Shekhar and Rahnev, 2018). IPV-PTSD individuals might present increased difficulties to attribute trustworthiness to relatively trustworthy avatars and would thus allocate less attention in encoding positive-valenced avatars.

Regarding the LPP component when decoding processing of relatively trustworthy avatars, we again observed differential group activity with decreased left angular gyrus activity in the IPV-PTSD group compared with non-PTSD controls. The left angular gyrus is involved in memory-retrieval (Seghier, 2013) and thus may be associated with increased difficulty in associating facial avatars that were presented with existing memories.

Electroencephalography findings first demonstrated that IPV-PTSD individuals react differently to dominance-related avatars and trustworthiness-related avatars. Encoding of dominance-related avatars leads to an overactivation of the limbic system in individuals with PTSD, which would probably be associated with increased hyperarousal symptoms of PTSD. In contrast, we observed the involvement of more attentional-related processing (i.e., measured in the aPFC), in response to relatively trustworthy avatars that appear as dysfunctional in IPV-PTSD individuals compared with non-PTSD controls.

Those alterations in IPV-PTSD individuals, in line with prior studies that consider how the misinterpretation of facial expression affects social judgment (Fide et al., 2019), might impact social functioning more generally. In our study, the fact that all of our participants also happen to be mothers raises the possibility that such alternations in information processing among IPV-PTSD affected women may be additionally associated with transgenerational transmission of these difficulties in emotion appraisal and thus social judgment to their children.

### Limitations

One potential limitation of the present study is related to the fact that we used a diagnosis of lifetime PTSD that based on assessment made 4 years prior, the latter having been based on the DSM-IV-TR criteria. In the present study, we were interested in the way the lifetime diagnosis of PTSD affected women’s evaluation of avatars subsequently. While we co-varied the presence of PTSD symptoms and their severity since the original psychiatric assessment, we were not able to account for the onset of comorbid psychopathology or for the possible effects of mental health treatment since the original assessment.

Moreover, exposure to IPV, which was measured retrospectively—and thus subject to greater error than prospective measurement, also varied significantly in type, age of onset, chronicity, repetitiveness, and degree of injury across subjects, and so the results given the sample size and distribution of life events may not be representative of a broader community sample. That being said, based on the literature, this study’s sample does appear to represent a typical and naturalistic representation of women with IPV-PTSD (Afifi et al., 2017; Machisa et al., 2017). With these points in mind, we note, nevertheless, that this is a study of cross-sectional associations such that we cannot assume causality but only speculate as to cause and effects with respect to maternal interpersonal violent trauma history and appraisal of facial avatars.

Finally, validation and normed measurement of the degrees of dominance and trustworthiness characteristics of the male facial avatars in a non-clinical sample had been done within a population of educated, primarily Caucasian American adults including both women and men (Oosterhof and Todorov, 2008; Todorov et al., 2008, 2013). While we assume that a Francophone Swiss metropolitan sample of women of childbearing age would rank these avatars similarly, validating measurement of their appraisal in this context was beyond the scope of the present study. Moreover, we were not able to find another study considering evoked potential in response to dominance- and/or trustworthiness-related avatars in an evaluation task.

Finally, it is a natural limitation of current laboratory EEG that participants can perceive it as obtrusive. In future studies, acquiring physiological measures through less obtrusive ways, such as ultra-wearable hearables (Goverdovsky et al., 2017), may improve this situation and provide a better way forward.

### Conclusion and Clinical Applications

The present study indicates that processing of threatening facial stimuli is in adult women with IPV-PTSD significantly differed from that of women without PTSD. This difference was measured by self-report measures (i.e., questionnaires, evaluation task) and observational measures, such as EEG. Moreover, these differences were measured across multiple timepoints during processing of stimuli and involved brain regions that are associated with emotion appraisal and regulation as well as memory.

These findings are likely to be useful in the development of clinical evaluation tools and treatment in the service of preventing revictimization of women who have experienced IPV. The present findings are thus relevant to public mental health, safety, and practice.

## Data Availability Statement

The original contributions presented in the study are included in the article/supplementary material. Further inquiries can be directed to the corresponding author/s.

## Ethics Statement

The studies involving human participants were reviewed and approved by Geneva University Hospitals and Faculty of Medicine. The patients/participants provided their written informed consent to participate in this study.

## Author Contributions

SRS and DS designed the study. VP and MV collected the data. VP and DM worked on data analyses. VP interpreted the data and wrote the main manuscript, with support of DM, DS, and AT. VP created figures and tables. All authors contributed to the article and approved the submitted version.

## Conflict of Interest

The authors declare that the research was conducted in the absence of any commercial or financial relationships that could be construed as a potential conflict of interest.

## Footnotes

1 https://facegen.com

2 http://tlab.princeton.edu/databases

3 https://sites.google.com/site/cartoolcommunity

4 http://www.bic.mni.mcgill.ca/brainweb
